# Does the Inclusion of Virtual Reality Games within Conventional Rehabilitation Enhance Balance Retraining after a Recent Episode of Stroke?

**DOI:** 10.1155/2013/649561

**Published:** 2013-08-18

**Authors:** B. S. Rajaratnam, J. Gui KaiEn, K. Lee JiaLin, Kwek SweeSin, S. Sim FenRu, Lee Enting, E. Ang YiHsia, Ng KeatHwee, Su Yunfeng, W. Woo YingHowe, S. Teo SiaoTing

**Affiliations:** Nanyang Polytechnic, School of Health Sciences (Allied Health) 569830, Singapore

## Abstract

This randomised controlled and double-blinded pilot study evaluated if interactive virtual reality balance related games integrated within conventional rehabilitation sessions resulted in more superior retraining of dynamic balance compared to CR after stroke. 19 subjects diagnosed with a recent episode of stroke were recruited from a local rehabilitation hospital and randomly assigned to either a control or an experimental group. Subjects in the control groups underwent 60 minutes of conventional rehabilitation while those in the experimental groups underwent 40 minutes of convention rehabilitation and 20 minutes of self-directed virtual reality balanced rehabilitation. Functional Reach Test, Timed Up and Go, Modified Barthel Index, Berg Balance Scale, and Centre of Pressure of subjects in both groups were evaluated before and on completion of the rehabilitation sessions. Results indicate that the inclusion of interactive virtual reality balance related games within conventional rehabilitation can lead to improved functional mobility and balance after a recent episode of stroke without increasing treatment time that requires more health professional manpower.

## 1. Introduction

The world is aging and chronic conditions such as stroke are more prevalent [1]. Global fatality from stroke had reduced in most countries, but there is an increased demand for poststroke rehabilitation services [2]. Novel therapy such as virtual reality, robot-aided therapy, and neuromuscular electrostimulation reported varied outcomes during rehabilitation. A review by Oujamaa and colleagues [3] found motor improvement reported in 4 studies and 6 reported functional improvement when mixed techniques of intensive task-oriented repetitive training using constraint induced therapy and bilateral task training were included in their rehabilitation programme in the first 6 months after stroke without any additional hours compared with experimental group. Laver and colleagues [4] found limited evidence to conclude that the use of virtual reality and interactive video gaming improved arm function and activities of daily living (ADL) function when compared with the same dose of conventional therapy. Moreover, conflicting evidence exists to support the transference of trained task from the virtual environment to the real world [5]. Sisto et al. [5] claim that despite virtual reality being able to systematically and in a serial fashion allow one to explore sensory, motor, and visual strategies that are important for movement, it may actually slow down recovery as performance gains are seldom transferred to performance of real-world tasks.

Poor postural control after stroke has a significant impact on a patient's ability to achieve independence in activities of daily living and gait [6–10]. Di Monaco and colleagues [11] reported that balance is one of the most significant predictors of functional independence after stroke (*β* = 0.37, *P* = 0.004). Poor balance moderately correlated (*r* = 0.581, *P* = 0.001) with poor ambulatory activity [12]. A recent literature review indicating that interventions incorporating balance exercise are effective in reducing the risk of falls [13]. Despite the above results, 73% of patients discharged from hospitals after stroke experienced a fall [8], indicating the importance of effective balance retraining during in-patient rehabilitation. 

Dejong et al. [14] reported a strong association between the amounts of physical therapy time patients received and their level of functional outcomes on discharge. More time spent on gait training was positively associated with better outcomes among patients with total knee arthroplasty (*β* = 0.160, *P* < 0.001) and after stroke (*β* = 0.204, *P* < 0.001). However, due to the worldwide shortage of health care professionals especially in the area of rehabilitation [15], patients after stroke were reported to receive an average of only 44 minutes (range: 42.6–45.4 minutes) of physical therapy a day [14]. Interactive virtual reality games create life-like three-dimensional environments that can be introduced within rehabilitation to facilitate patients to regain their bodily function with minimal assistance from therapist [4, 5]. Virtual reality games were found to be feasible and beneficial when introduced to an elderly patient who experienced recurrent falls [16]; they decreased Community Balance and Mobility Scale scores and improved reaction time among 12 healthy older adults after 10 weeks of training [17], and 27 moderate to severe traumatic brain injured individuals reported improvement in balance confidence and function after participating in a virtual reality delivered balance exercise programme for 6 weeks [18]. However, no randomised controlled trial has evaluated the efficacy of integrating interactive virtual reality balance related games within conventional rehabilitation sessions to retrain dynamic balance and motor recovery among patients after stroke. 

This double-blinded and randomised pilot study evaluated the effectiveness of introducing low cost interactive virtual reality balance related games within a conventional rehabilitation programme. The focus was to evaluate if including virtual reality balance related games during inpatient rehabilitation sessions without increasing therapy time would contribute to balance recovery among in-patients after a recent episode of stroke.

## 2. Material and Method

The study was approved by Ang Mo Kio Hospital in Singapore and the Projects Committee of the School of Health Sciences (Allied Health) at Nanyang Polytechnic, Singapore (SHS/2009-2010/02/PT).

### 2.1. Subjects

A total of 19 in-patients (age: M = 61.6; SD = 7.8) from the community rehabilitation hospital participated in this study which was conducted over two years. The inclusion criteria were all subjects who had recently experienced a first onset of stroke (days after stroke; mean = 14.85 ± 6.8) and were graded to have moderate disability (mRS Grade 3) or moderate severe disability (mRS Grade 4) using the 6-point ordinal hierarchical Modified Rankin Scale. mRS is the most commonly used functional measure in stroke trials and takes around 5 minutes to complete. They also were evaluated to be medically stable and had a Mini-Mental State Examination score of greater than 23 and thus understood how to safely use virtual reality gaming consoles (Table 1). Exclusion criteria included terminal diseases, uncontrolled hypertension and angina, and severe spatial neglect or visual impairments. All 19 subjects met the inclusion and exclusion criteria. 

### 2.2. Experiment Protocol

Random Allocation Software assigned subjects to either 60 minutes of conventional rehabilitation group (control) or the experimental group which received 40 minutes of conventional rehabilitation with 20 minutes of interactive virtual reality balance related games (Experimental). Subjects in the experimental group were introduced to either a Nintendo Wii-Fit or Microsoft Kinect game console system during rehabilitation. All subjects received 15 sessions of in-patient rehabilitation during their hospital stay. 

**Figure 1:**
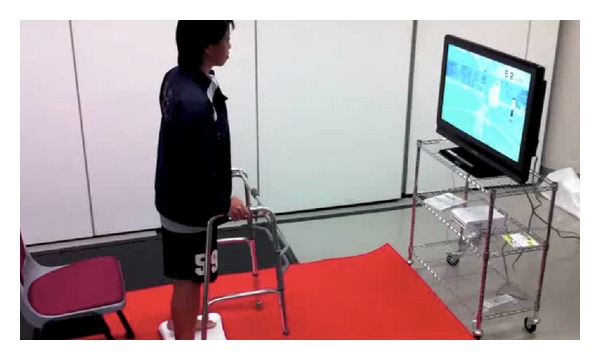
Subject standing on the Nintendo Wii-Fit Balance Board attached to a TV screen.

**Figure 2:**
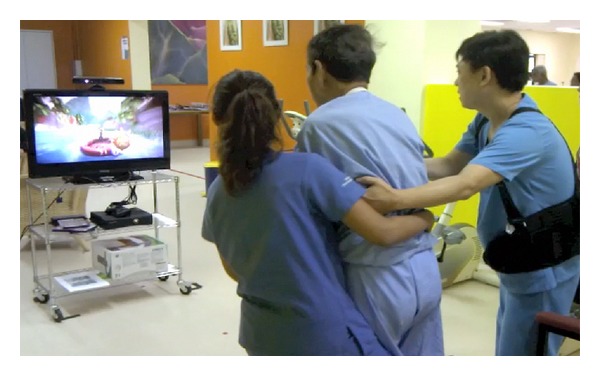
Subject responding to game console images from Microsoft Kinect.

**Figure 3:**
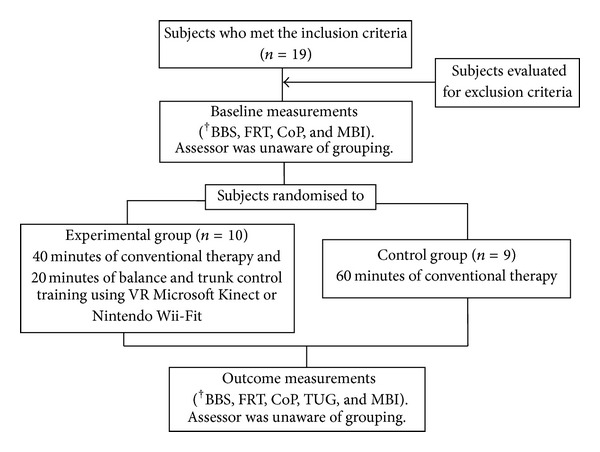
Flow chart of the experiment procedure. ^†^BBS = Berg Balance Scale, FRT = Functional Reach Test, CoP = Centre of Pressure using Nintendo Wii-Fit, TUG = Timed Up and Go, MBI = Modified Barthel Index.

The Nintendo Wii-Fit programme required subjects to shift their weight during standing in response to the game (Figure 1). The Microsoft Kinect game system required them to constantly change their centre of mass in both sitting and standing (Figure 2).  Figure 3 illustrates the experimental procedure.

### 2.3. Outcome Measures

One researcher who was not involved in the interventions and was blinded to the allocation of subjects to their groups' evaluated both groups Functional Reach Test (FRT), Timed Up and Go (TUG), Berg Balance Scale (BBS), Centre of Pressure (CoP), and Modified Barthel Index (MBI) before and after the intervention period. FRT is a quick and easy dynamic test of anterior-posterior stability evaluated when subjects perform one arm forward reach task. The test has an excellent test-retest (*r* = 0.89) and interrater reliability (*r* = 0.98) and strongly correlated (*r* = 0.78) with BBS [19].

TUG assesses functional mobility and claims to predict those at risk of falls if their timing is greater than 14.7 sec [20]. Furthermore, TUG has a strong correlation (*r* = −0.79) with Barthel Index [20]. BBS evaluates subjects' ability to maintain balance while performing 14 everyday functional tasks. It has a good internal consistency, test-retest and interrater reliability (*r* ≥ 0.75), and construct validity (*r* ≥ 0.60) with Modified Barthel Index [21]. 

Center of Pressure (CoP) sway was measured using the Nintendo Wii-Fit Board. This device evaluated balance and was reported to be comparable with force platforms with a high test-retest reliability (ICC = 0.77–0.89) [22]. The Modified Barthel Index (MBI) assessed the level of one's functional independence and reported to have a high interrater reliability and internal consistency [23]. 

### 2.4. Data Analysis

Data collected from this pilot study were evaluated and found not to be normally distributed. Thus, data were evaluated with Mann-Whitney *U* tests for between-group differences at baseline and after intervention. Wilcoxon sign ranked test determined within-subject changes between the baseline and post-intervention values in both groups. Data were analysed with intent-to-treat analyses. The level of significance was set at *P* < 0.05. The data were analysed using SPSS (version 17.0 for Windows, SPSS Inc., Chicago, IL, USA).

## 3. Results

### 3.1. Outcome Measures between Groups

Mann-Whitney *U* tests found no significant differences in baseline measure of all outcomes measures in both groups (TUG: *U* = 18, *P* = 1.000; BBS: *U* = 0.5, *P* = 0.570; COP: *U* = 50, *P* = 0.857; FRT: *U* = 44.5, *P* = 0.968; MBI: *U* = 13.5, *P* = 0.485). This implied both groups baselines were similar, and thus comparison after intervention would reveal intervention effect. There was a significant difference in FRT scores between the experimental and control groups after 15 sessions of rehabilitation (*U* = 16, *P* = 0.017). There were no statistically significant differences in all other outcome measures after intervention between the control and experiment groups (Table 2). FRT was significantly correlated with BBS (*r* = 0.807, *P* = 0.028).

### 3.2. Outcome Measure within the Experiment Group

The introduction of low cost interactive virtual reality balanced related games within a conventional rehabilitation programme significantly improved TUG, FRT, and MBI scores (Table 2). 

### 3.3. Outcome Measure within Control Group

15 sessions of convention therapy resulted in significant improvements in TUG and MBI scores (Table 2).

## 4. Discussion 

This randomised controlled and double-blinded pilot study found that introducing interactive virtual reality balance related games within the standard treatment time of conventional therapy sessions over 15 sessions was equally effective as conventional therapy alone to retrain balance after a recent episode of stroke. The inclusion of virtual reality games without providing any additional therapy time or professional intervention to the experimental group significantly improved their Functional Reach Test mean scores by more than the double compared with the control group (experimental group's mean = 5.1020 ± 2.9144 versus control group's mean = 1.5544 ± 3.54079). 

Smith et al. [19] reported that Functional Reach Test is a dynamic test of one's anterior-posterior stability and strongly correlated with Berg Balance scores. Our results concur with their findings. We also found a strong correlation (*r* = 0.807, *P* = 0.028) between these two outcome measures. Our findings also support the hypothesis that participation in interactive virtual reality balance related games was more effective than conventional exercises to maintain postural stability during walking as it replicates the load-unloading sway strategy at the hip [24]. Moreover, the elderly rely more on hip control to regulate their balance during walking compared to young adults [25]. Cumulatively, the results indicate that interactive virtual reality balance related games within conventional rehabilitation programmes retrains one's Centre of Pressure in different directions, ranges and speeds frequently and elicits effective ankle and hip postural control strategies to maintain functional mobility affected with increase age [24]. 

The results of this robust pilot study conducted on patients after a recent stroke extends finding found among “healthy” older adults that virtual reality games placed greater demands and challenges on one's neuromuscular system compared to conventional balance programmes [26]. Our results add further evidence to support the already large pool of published literature that report that interactive virtual reality balance related games can improve balance and mobility after stroke and in the elderly [26–28], participants with balance impairment [29], healthy older persons [24, 30], and middle-aged adults [31]. We found introducing subjects who experienced a recent episode of stroke to virtual reality balance related games within their rehabilitation programme which resulted in 33% more recovery of functional mobility (a.k.a. TUG score) and 28% more independence in activities of daily living (a.k.a. MBI score) than those who received only conventional therapy. We hypothesise that virtual reality balance related games designed to mimic conventional rehabilitation goals of retraining timely, goal directed, and rapid postural weight shift led to patients in the experimental group experiencing greater improvement in functional mobility and independence in their activities of daily living. Deutsch et al. [27] and Sugarman et al. [28] reported that persons introduced to Nintendo Wii-Fit programme increased their gait speed, walking endurance, balance, and dual tasks mobility scores. Sugarman et al. [28] reported that introducing virtual reality games to an 86-year-old patient five weeks after stroke resulted in a 10-second improvement in TUG. 

Conventional balance training during rehabilitation led to poor engagement and lack of interest by patients due to repetitive practice of the same exercises [32, 33]. However, Meldrum et al. [29] found patients after stroke enjoyed and preferred virtual reality balance games to conventional therapy. Creating a degree of engagement and fun motivated and improved their compliance [29] and increased their attention span to spend more time on their rehabilitation programme [24, 32]. Interactive virtual reality balance related games also provided immediate visual feedback of their performance and empowered them with a sense of control over their recovery as they engaged in more self-practice in a fun manner [32, 34–36]. A recent Cochrane review found exposing older adults to multiple exercises types had greater impact on improving balance compared to one exercise type [37]. Virtual reality games incorporate multiple actions within a game and exposed one to different levels of difficulties which may explain the improvement in dynamic stability and balance. 

The finding of this pilot study could encourage physical therapist to introduce interactive virtual reality games during rehabilitation to those who have physical and cognitive abilities to take responsibility for their recovery. Properly selected virtual reality games can be socially engaging, entertaining, and readily accepted and enjoyed by most patients [38, 39]. Physical therapist should work with interactive digital media professions to design games that are aligned with therapeutic goals and do not encourage adaptive postural reactions [38]. 

Moreover, the games can be performed safely and effectively at any time and at any location including within and outside rehabilitation sessions with older adults [24, 28], those with mild Alzheimer's disease [40], and community-dwelling fallers over 70 years of age [41]. We recommend that physical therapist carefully selects patients who will benefit from virtual reality games and assigns a general care staff or family member to be present for their safety when performing virtual reality enhanced rehabilitation. 

We found that participation in 20 minutes of interactive virtual reality balance related games integrated within a rehabilitation session frees up 33% of physical therapist's time. Physical therapist could utilise the time gain with the introduction of virtual reality games with other patients that require more intensive therapy. Interactive virtual reality games should be welcome within rehabilitation session as this pilot study found it improved efficacy and efficiency of busy and scarce physical therapy professionals.

*Limitations.* The main limitation of this pilot study was the small sample size. The results could be considered to be false positive. Post hoc evaluation indicated that future studies should involve a minimum of 50 subjects in each group to have a large effect size. Moreover, the selection of patients in this study reflects those with moderate disability based on the Modified Rankin Scale. Thus, the results cannot be generalised to rehabilitation of all patients after stroke. 

Furthermore, this study did not evaluate the long-term effects of interactive virtual reality balance related games within rehabilitation. “Correct” motor performance could be achieved by introducing motion sensors especially during practice sessions outside therapy time. This is important as early stage rehabilitation should maximise neuroplasticity by facilitating return of “normal” motor recovery rather than compensatory movement patterns. 

## 5. Conclusion

Interactive virtual reality balance related games are a viable adjunct to include within conventional rehabilitation sessions without increasing treatment time or requiring more professional attention. It was well received by in-patients and resulted in improvement in balance. Well-designed games based on therapeutic principles will empower patients to be involved in their rehabilitation while freeing up scarce therapist's time. A larger scale randomised control trial is required to confirm our findings and further evaluate the long-term effects of introducing virtual reality games as self-initiated exercises within the rehabilitation protocol of patients after stroke.

## Figures and Tables

**Table 1:** Characteristics of subjects in the experiment and control groups.

	Control group (*n* = 9)	Experimental group (*n* = 10)
Age in years, mean (SD)	65.33 (9.59)	58.67 (8.62)
Gender (male/female)	3/6	4/6
Side (left/right)	4/5	4/6
Type of stroke (infarct/haemorrhage)	8/1	8/2
Modified Rankin Scale (Grade 3/Grade 4)	5/4	6/4
Days after stroke	15.2 (6.3)	14.7 (7.5)

**Table 2:** Postintervention and pre-/postdifferences between and within groups.

	Postintervention differences between groups		Pre-/postdifferences within experimental group		Pre-/postdifferences within control group	
	Mann-Whitney *U* test	*P* value (2 tailed)	Wilcoxon sign ranked *Z*-score	*P* value (2 tailed)	Wilcoxon sign ranked *Z*-score	*P* value (2 tailed)
TUG	24	0.394	−2.201	0.028*	−2.201	0.028*
BBS	3	0.400	−1.604	0.109	−1.604	0.109
CoP	8.5	0.400	−0.552	0.581	−1.069	0.285
FRT	16	0.017*	−2.803	0.005*	−1.363	0.173
MBI	24	0.394	−2.207	0.027*	−2.201	0.028*

*P < 0.05.
